# Homeostatic Regulation of *Salmonella*-Induced Mucosal Inflammation and Injury by IL-23

**DOI:** 10.1371/journal.pone.0037311

**Published:** 2012-05-18

**Authors:** Muyiwa Awoniyi, Samuel I. Miller, Christopher B. Wilson, Adeline M. Hajjar, Kelly D. Smith

**Affiliations:** 1 Department of Pathology and Program in Molecular and Cellular Biology, University of Washington, Seattle, Washington, United States of America; 2 Departments of Medicine, Genome Sciences and Microbiology, University of Washington, Seattle, Washington, United States of America; 3 Department of Immunology, University of Washington, Seattle, Washington, United States of America; 4 Department of Comparative Medicine, University of Washington, Seattle, Washington, United States of America; New York University, United States of America

## Abstract

IL-12 and IL-23 regulate innate and adaptive immunity to microbial pathogens through influencing the expression of IFN-γ, IL-17, and IL-22. Herein we define the roles of IL-12 and IL-23 in regulating host resistance and intestinal inflammation during acute *Salmonella* infection. We find that IL-23 alone is dispensable for protection against systemic spread of bacteria, but synergizes with IL-12 for optimal protection. IL-12 promotes the production of IFN-γ by NK cells, which is required for resistance against *Salmonella* and also for induction of intestinal inflammation and epithelial injury. In contrast, IL-23 controls the severity of inflammation by inhibiting IL-12A expression, reducing IFN-γ and preventing excessive mucosal injury. Our studies demonstrate that IL-23 is a homeostatic regulator of IL-12-dependent, IFN-γ-mediated intestinal inflammation.

## Introduction

Non-typhoidal *Salmonella* is the most common cause of bacterial-related deaths in the United States [1], and *S. enterica* serovar Typhimurium is the most frequent isolate. During the infection, *Salmonella* utilizes type III secretory systems to introduce bacterial effector proteins into the host cells contributing to acute inflammatory responses [2], which result in enterocolitis and diarrhea [3]. We are interested in better understanding host innate immune factors that contribute to resistance against *Salmonella* and control the quality and quantity of the inflammatory response to this pathogen. In this study, we focus on the role of IL-12 and IL-23 in regulating host resistance and mucosal inflammation during acute *Salmonella* infection. Individuals with deficiencies in IL-12 and IL-23 signaling, due to defects in either *IL12Rβ1* or *IL12/IL23p40* genes are prone to developing persistent *Salmonella* infections [4]. Mice with deficiencies in IL-12 and IL-23 are also more susceptible to systemic *Salmonella* infection [5] [6].

IL-12, a heterodimeric cytokine consisting of p35 and p40 subunits, is produced during *Salmonella* infection by dendritic cells and macrophages, and regulates T-cell and NK cell IFN- γ production [7]. During *Salmonella* infection in mice, IFN-γ activates macrophages, and in the absence of IL-12, IFN-γ production is reduced and susceptibility to infection is increased [8]. Both IL-12 and IFN-γ help provide strong protective immunity against intracellular pathogens, such as non-typhoidal *Salmonella*, Mycobacterium species [9], influenza virus, *Francisella tularensis*, and *Yersinia pestis* [10]. IL-23, a heterodimer of p19 and p40 [11], is a key cytokine for the development and function of Th17 cells, and contributes to the production of IL-17 and IL-22 by T-cells and lymphoid tissue inducer-like cells [12], [13]. IL-23, IL-22 and IL-17 help control infections by extracellular pathogens *Candida albicans, Citrobacter rodentium, and Mycoplasma pneumonia*, and more recent data have also shown roles in inflammatory responses elicited by *Salmonella enteritidis, Chlamydia muridarum, Pseudomonas aeuginosa, and Mycobacterium bovis* [14], [15], [16], [17], [18], [19]. Thus the nature of the pathogen (extracellular vs. intracellular) may not be the sole determinant for IL-23 mediated protective immunity, and other factors, such as site of infection (systemic vs. mucosal) may also influence immunity and the roles of IL-12 and IL-23.

Systemic and typhoidal mouse models of *Salmonella* infection indicate that IL-23's function is largely masked by IL-12 [6] and that IL-23 enhances protection against systemic *Salmonella* infection primarily through IL-22 [6] and to a lesser extent IL-17A [20]. Because mice are highly resistant to intestinal colonization by *Salmonella* and the murine typhoidal infection model does not evoke the prominent intestinal inflammation that is typically present in humans and larger animals, it is possible that the role of IL-23 is less important in mice [6]. In order to address this possibility, the role of IL-23 was analyzed in an acute colitis model in mice, which demonstrated that IL-23 was required for cecal inflammation, IL-17A production and neutrophil recruitment [14]. The γδ-T cells were the primary producers of IL-17A and cellular target for IL-23 [14]. The roles of IL-12 and its interactions with IL-23 during acute *Salmonella* induced enterocolitis have not been investigated.

In this study, we use the streptomycin pretreatment model of *Salmonella*-induced acute enterocolitis [21] to define the roles IL-12 and IL-23 in regulating host resistance and intestinal inflammation during acute *Salmonella* infection. Our data show that IL-12 promotes the production of IFN-γ, which is largely produced by NK cells during acute infection [22]. IFN-γ is required for resistance against *Salmonella* and also for induction of intestinal inflammation and epithelial cell injury. In contrast, IL-23 limits inflammation and mucosal injury. In conclusion, interactions between IL-12 and IL-23 help protect against mucosal *Salmonella* infection, limit IFN-γ-mediated intestinal injury, and maintain epithelial homeostasis.

## Results

### IL-12 and IL-23 control gut colonization and systemic spread during oral *S*. Typhimurium infection

**Figure 1:**
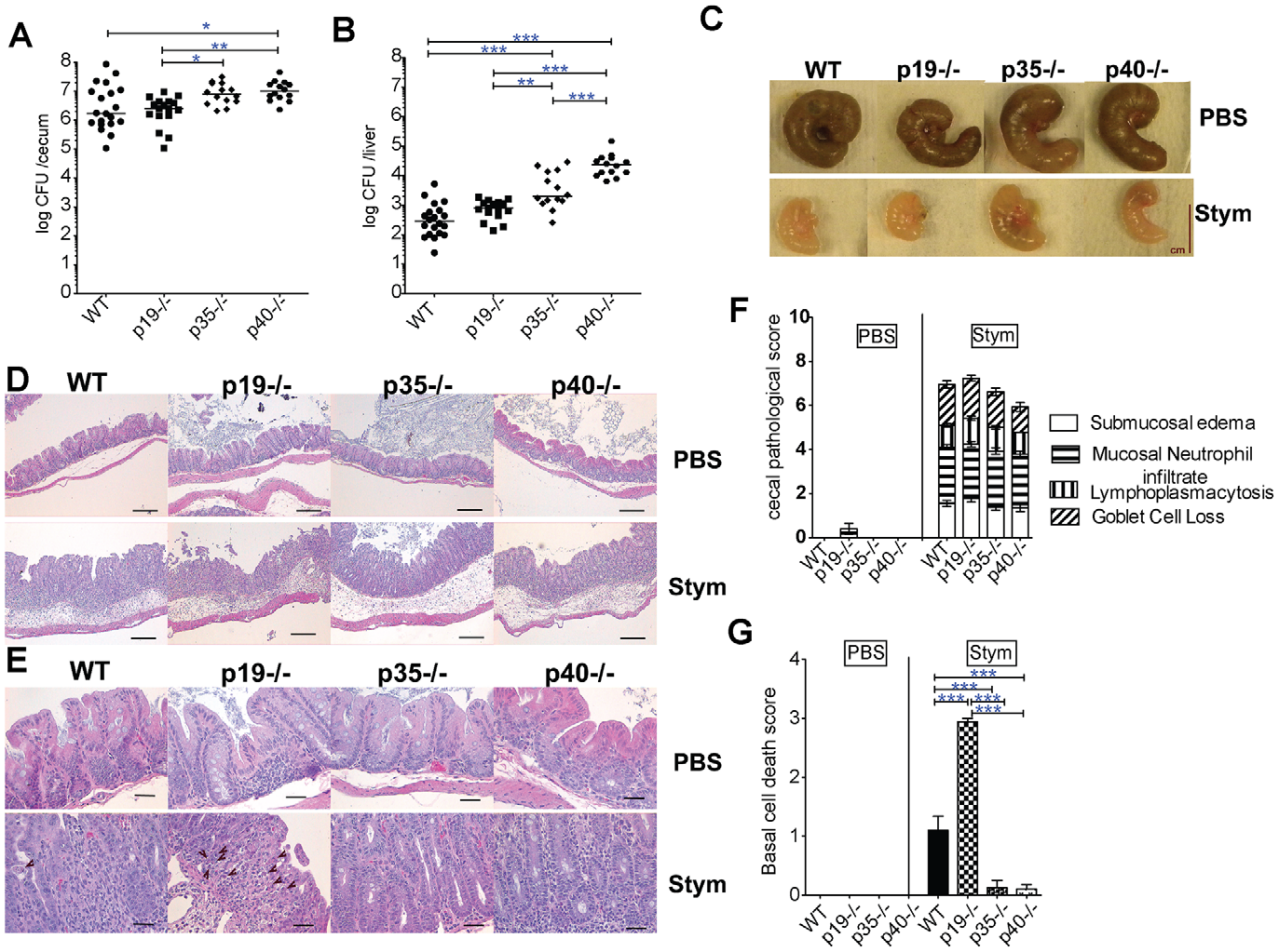
IL-12 and IL-23 are required for resistance against S. Typhimurium (Stym). WT, p19^−/−^(no IL-23), p35^−/−^(no IL-12), and p40^−/−^ (no IL-12/-23) mice were orally infected with 1×10^3^ CFU of *S*. Typhimurium 1 day after 20 mg streptomycin pretreatment. (A) Cecum and (B) liver were collected from infected mice 3 days post infection. Organs were weighed, homogenized and plated to determine CFU/organ. Bars represent the median bacterial load. Pooled data from 3 separate experiments are shown in A and B. (C) Representative ceca are shown for each group at 3 d after oral inoculation with PBS (mock) or Stym. A one centimeter bar indicates the magnification. (D) 10X and (E) 40X magnification of histopathology of H&E stained cecal sections of WT, p19^−/−^, p35^−/−^, and p40^−/−^ mice 3 d after infection. Bar in (D) represents 100 micron and (E) 20 micron. Notched arrow-heads in (E) indicate cell death. (F) Blinded scoring was performed on H&E-stained cecal sections. The inflammatory score equals the sum of the separate categories (edema, PMN infiltration, lymphoplasmacytosis, and goblet cell loss). (G) H&E stained cecal sections from WT, p19^−/−^, p35^−/−^, and p40 mice 3 d after infection were blinded and qualitatively assessed for cell death: 0 (no significant), 1 (focal), 2 (mild), 3 (severe) in the basal epithelium. At least 3 mock treated animals and at least 13 infected mice from each group from 3 separate experiments were scored in 1F and 1G. Mean values ± SE are shown. Statistical significance was determined using the one way ANOVA test with Bonferroni post test (A, B, F and G). *: p<0.05, **: p<0.01, ***: p<0.001.

Streptomycin pretreated C57BL/6, p19^−/−^, p35^−/−^ and p40^−/−^ mice were infected orally with a low dose of *S*. Typhimurium (10^3^ CFU) and bacterial burden was measured in the cecum and liver 3 days later (Figure 1A and B). IL-12 deficient mice (p35^−/−^) had elevated CFU in the liver relative to WT and had elevated counts in both the cecum and liver relative to IL-23 deficient mice. In contrast, mice deficient in both IL-12 and IL-23 (p40^−/−^) had elevated CFU in the cecum and liver when compared to WT or p19^−/−^ mice (Fig. 1A and B), and elevated liver CFU when compared to p35^−/−^ mice. These results suggest that loss of IL-12 alone results in increased early systemic dissemination, and that the additional loss of IL-23 increases cecal colonization, and further increases systemic spread, as seen in previous studies [6]. The loss of IL-23 alone did not affect gut colonization or systemic spread, as bacterial burden in p19^−/−^ mice was not significantly different from WT mice. Similar findings were observed for the p19^−/−,^ p35^−/−^ and wild-type C57BL/6 mice that were congenic for the *Slc11a1^G169/G169^* resistant allele, although the overall bacterial burden in the liver was lower in these mice, consistent with the protective role of Slc11a1^G169^ (Fig. S1).

### IL-12 family members differentially regulate cecal inflammation and injury

**Figure 2:**
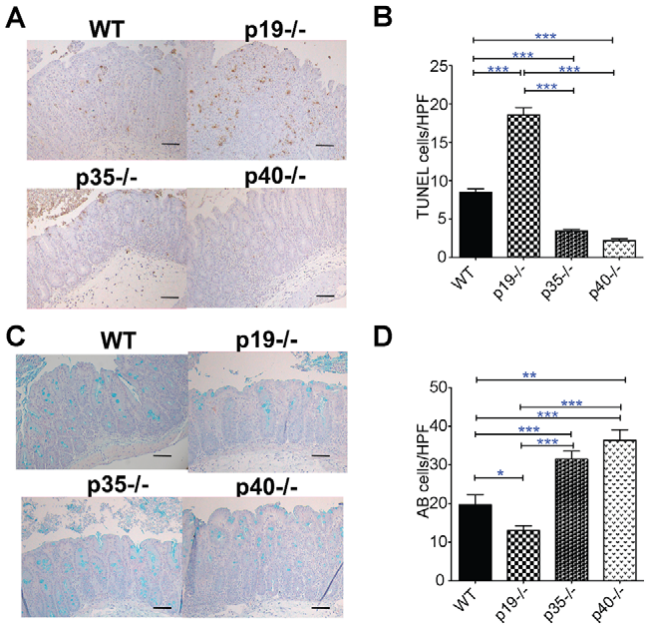
Loss of IL-23 in the context of functional IL-12 results in increased epithelial cell death and loss of goblet cells. (A) 20X magnification of TUNEL-positive (brown) or (C) Alcian Blue (blue) cells of representative infected cecal sections. The bar in (A) and (C) represents 20 micron. The mean ± SE of TUNEL (B) positive cells per high-power field was quantified from cecal sections WT (n = 4), p19^−/−^ (n = 5), p35^−/−^ (n = 6) and p40^−/−^ (n = 3) mice. The mean ± SE Alcian Blue positive cells were quantified per high-power field from cecal sections of the same mice (n = 3 per group). Statistical significance for (B and D) was determined using the one way ANOVA followed by Bonferroni post test. *: p<0.05, **: p<0.01, ***: p<0.001.

Gross examination of the ceca on day 3 following *S*. Typhimurium infection revealed that all ceca were small, thick and pale compared to mock treated controls (Fig. 1C). Infected ceca from p35^−/−^ and p40^−/−^ mice were slightly larger and had more visible cecal contents relative to WT and p19^−/−^ ceca suggesting less inflammation in the former groups. Histological analysis revealed similar levels of inflammation amongst the various groups (Fig 1F, with representative sections shown in 1D and 1E). Although not part of the inflammatory score, we noted that some mice had prominent numbers of apoptotic bodies in the lamina propria usually near the base of the epithelial crypts (arrows in Fig. 1E). Quantification of the histologic cell death revealed that p19^−/−^ mice had significantly more dead cells within the lamina propria compared to the other mice (Fig. 1G). To confirm our histological observations, we performed TUNEL staining, a common method for detecting DNA fragmentation that occurs during apoptotic cell death. Compared to *S*. Typhimurium infected WT mice, p19^−/−^ mice had more, and p35^−/−^ and p40^−/−^ mice had fewer TUNEL-positive cells (Fig. 2A and B). Conversely, the number of goblet cells, an indicator of epithelial cell differentiation, was decreased in the p19^−/−^ and increased in p35^−/−^ and p40^−/−^ mice relative to WT and p19^−/−^ mice, uncovering a novel role for IL-12 and IL-23 in regulating gut epithelial responses to *Salmonella* infection (Fig. 2C and D). Taken together, our data indicate that loss of IL-23 resulted in increased *Salmonella* induced epithelial cell death that required functional IL-12.

### Differential contribution of IL-17A and IL-22 to Salmonella induced inflammation and injury

**Figure 3:**
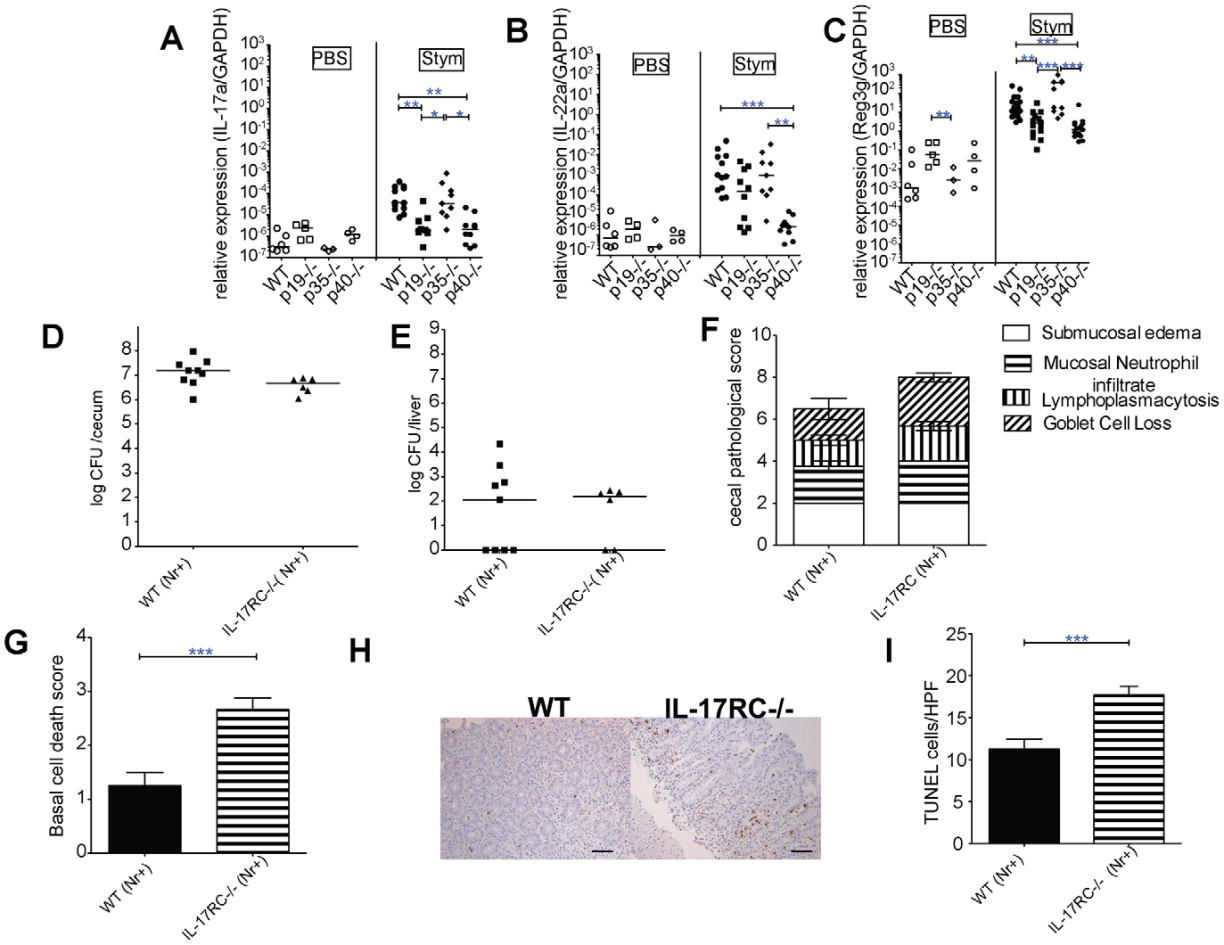
IL-17 has cytoprotective activity during Salmonella induced injury. (A) Changes in IL-17A, (B) IL-22 (C) Reg3γ gene expression elicited 3d after oral PBS (mock) or *S*. Typhimurium (Stym) infection in the ceca of WT, p19^−/−^, p35^−/−^, p40^−/−^ mice were measured by qPCR. Data are expressed as the ratio of mRNA levels of the gene of interest divided by GAPDH expression from the same RNA. Pooled data from 3 separate experiments are shown. (D) Cecum and (E) liver were collected from IL-17RC^−/−^ and C57BL/6 mice congenic for *Slc11a1* ^G169/G169^ three days post infection. Organ homogenates were diluted and plated to determine CFU/organ. Bars represent the median bacterial load from two experiments. (F) Histological changes and (G) basal cell death at 3 d post-infection from infected WT and IL-17RC^−/−^ H&E stained cecal sections were done as described in Fig. 2. (H) 20× magnification of TUNEL-positive (brown) cells of representative infected cecal sections. Bar in (H) represents 50 micron. (I) TUNEL positive cells were quantified per high-power field in the cecal sections from a minimum of 3 infected mice and two experiments. Statistical significance was determined using one way ANOVA with Bonferroni post test for A-C, Mann Whitney for D&E, and unpaired Student's t-test for F, G & I. *: p<0.05, **: p<0.01, ***: p<0.001.

We next hypothesized that downstream targets of IL-23 may protect the epithelium from apoptosis. We therefore examined gene expression in cecal tissue for IL-17A, IL-22, and Reg3γ. IL-17A expression correlated strongly with IL-23 genotype; IL-17A was induced to similar levels in WT and p35−/− mice, and not induced in p19^−/−^ and p40^−/−^ mice (Fig. 3A). In contrast, induction of IL-22 was only absent in p40^−/−^ mice (Fig. 3B), while Reg3γ, an antimicrobial peptide that is regulated by IL-22 [16], was reduced in both p19^−/−^ and p40^−/−^ mice (Fig. 3C and 3B).

To test whether IL-17 or IL-22 may have a cytoprotective role during *S*. Typhimurium infection in the cecum, we infected WT and IL-17RC^−/−^ mice (C57BL/6 *Slc11a1* ^G169/G169^ background) and found that similar to p19^−/−^ mice (Fig 1A and B), the loss of IL-17RC did not affect cecal or liver CFU (Fig. 3D and E). The IL-17RC gene is expressed highly in the small intestine and colon in both mice and humans, and in particular by murine colonic epithelial cells [23], [24], [25]. IL-17RC appears to be required for both IL-17A and IL-17F signaling [25]. The ceca from infected mice showed comparable levels of inflammation (Fig. 3F). However, cell death was increased in IL-17RC^−/−^ mice (Fig 3G), which was confirmed by TUNEL staining (Fig. 3H and 3I), and suggests that IL-17 may have a cytoprotective effect on cecal epithelium similar to that observed for IL-23. We also infected IL-22^−/−^ C57BL/6 mice and found that IL-22 did not affect cecal colonization or systemic spread of *S*. Typhimurium (Fig. S2A–B). However, IL-22^−/−^ mice had significantly less inflammation and fewer TUNEL-positive cells than C57BL/6 controls (Fig. S2C–D), suggesting that IL-22 contributes to acute cecal inflammation. Decreased cell death in the IL-22−/− mice correlated with the decreased inflammation.

### Preservation of TNF, IL-6, IL-1B and Nos2 expression correlates with protection against *Salmonella* infection

**Figure 4:**
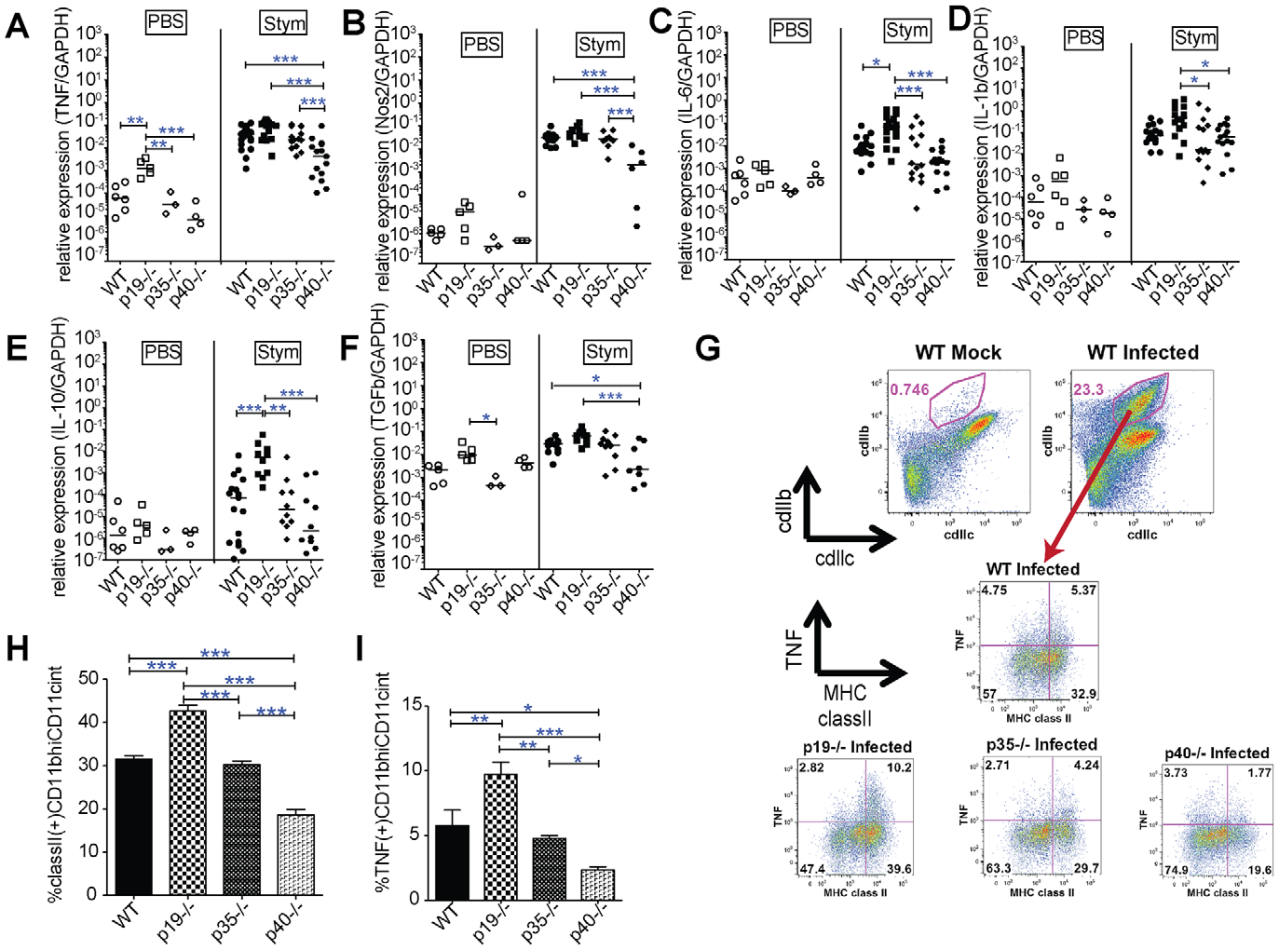
IL-23 limits IL-12 dependent pro-inflammatory cytokine production and the activation of gut mononuclear phagocytes. (A) TNF, (B) Nos2, (C) IL-6, (D) IL-1β, (E) IL-10 and (F) TGF-β cecal gene expression at 3 d after oral *S*. Typhimurium or mock infection of WT, p19^−/−^, p35^−/−^, p40^−/−^ mice was determined by qPCR. Data are expressed as the ratio of mRNA levels of the gene of interest divided by GAPDH expression from the same RNA. Bars show median values and the data are pooled from 3 separate infections. (G) Following mock or *Salmonella* infection, lamina propria (LP) cells were isolated from the cecum and colon of C57BL/6 mice as detailed in Materials and Methods section. LP cells were treated for 4 hrs *ex vivo* with BFA, followed by surface staining with CD11c, CD11b and class II MHC, followed by intracellular TNF. The CD11b^hi^ CD11c^+^ cell population (red gates) were the primary TNF producers. TNF and class II MHC expression by the CD11b^hi^ CD11c^+^ cell populations is shown for representative animals in the lower panels. Quantification of CD11b^hi^/CD11c^+^ positive cells for (I) MHC classII^hi^ expression and (J) MHC classII^hi^ TNF^+^ populations. Statistical significance was determined using one way ANOVA followed by Bonferroni post test. *: p<0.05, **: p<0.01, ***: p<0.001.

We determined whether the expression of selected genes correlated with protection against *Salmonella* infection (Fig. 4A–D). TNF and Nos2 were decreased in the p40^−/−^ relative to all other mice (Fig. 4A and B); basal levels of TNF were increased in mock-infected p19−/− mice although levels were substantially lower than infected mice. In addition, p19−/≤ expressed more IL-6 than each of the other mice (Fig. 4C), and more IL-1B than the p35−/− and p40−/− mice (Fig. 4D). Anti-inflammatory cytokines, IL-10 and TGF-β (Fig 4E and F) showed similar patterns of expression as the pro-inflammatory cytokines, suggesting that decreased anti-inflammatory cytokine expression did not explain the increased inflammation and injury.

TNF and Nos2 expression appear to be critical for limiting *Salmonella* growth in mice, as evidenced by the marked susceptibility of p40−/− mice to infection. TNF and Nos2 producing dendritic cells are critical mediators of host defense against the intracellular bacterium *Listeria monocytogenes* [26]. To identify cells that were producing TNF, we isolated and examined lamina propria cells from mock and *Salmonella* infected C57BL/6 mice. *Salmonella* infection resulted in a marked expansion of CD11b^hi^, CD11c^+^ cells (Fig. 4G). During *S*. Typhimurium infection, these cells upregulated class II MHC and were the primary producers of TNF; neutrophils (CD11b^hi^, Ly6g^hi^ CD11c^null^ MHC classII^null^ cells) also contributed to a lesser extent (data not shown). Within the CD11b^hi^, CD11c^+^ cell populations, the proportion of MHC class II positive and the TNF/MHC class II double positive populations were significantly increased in p19^−/−^ and decreased in p40^−/−^ relative to the other mice (Fig. 4I and J). These data indicate that loss of IL-23 promotes the accumulation of TNF producing cells when functional IL-12 is present, and limits their accumulation when IL-12 is absent.

### IL-23 regulates IFN-γ mediated enterocolitis in an IL-12 dependent manner

**Figure 5:**
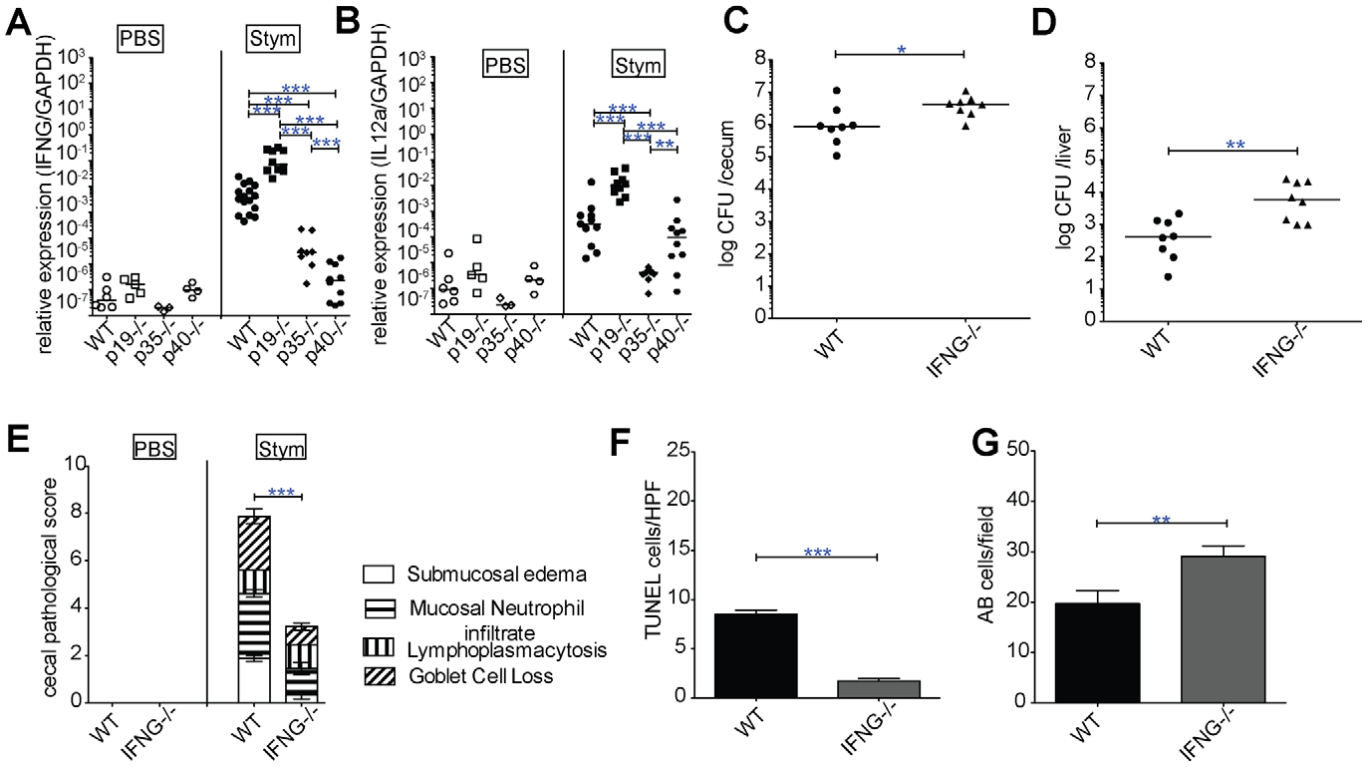
IL-23 inhibits *Salmonella* induced IL-12 dependent IFN-γ mediated inflammation. (A) IFN-γ, and (B) IL-12a (IL-12p35) cecal gene expression at 3 d after oral WT *Salmonella* infection +/− mock treated mice from WT, p19^−/−^, p35^−/−^, p40^−/−^ mice was determined by qPCR. Bars show the median and the data are pooled from 3 separate infections. (C) Cecum and (D) liver were collected from WT and IFN-γ^−/−^ 3 days post infection. Organ homogenates were diluted and plated to determine CFU/organ. Bars represent the median bacterial load and the data are pooled from 2 separate infections. (E) Histologic changes were quantified on H&E-stained cecal sections as described in Fig. 2. (F) TUNEL and (G) AB positive cells were quantified per high-power field in the ceca of at minimum 3 infected mice/group. Results are expressed in mean ± SE. Statistical significance was determined using one way ANOVA followed by Bonferroni post test for A and B, the Mann-Whitney test was used for C and D, and unpaired Student's t-test was used for E-G. *: p<0.05, **: p<0.01, ***: p<0.001.

Increased expression of IL-6 and IL10 in the ceca of infected p19−/− mice was dependent on functional IL-12 (Fig. 4C and E). Because some of the consequences of IL-23 deficiency were dependent on the functional status of IL-12, we examined the expression of IFN-γ, a major downstream target of IL-12, in cecal tissue of infected mice. Unexpectedly, the loss of IL-23 also led to significantly elevated IFN-γ message; as expected p35^−/−^ and p40^−/−^ mice had decreased levels of IFN-γ relative to WT mice (Fig. 5A). To better characterize the mechanism for IL-23 inhibition of IFN- γ^−/−^ we analyzed IL-12p35 expression (Fig 5B). IL-12p35 expression was increased in p19^−/−^ relative to WT mice and directly correlated with IFN-γ levels. This suggests that IL-23 inhibits IFN-γ in an IL-12p35 dependent manner. To further explore the role of IFN-γ in cecal inflammation and injury, we infected IFN-γ^−/−^ mice with *S.* Typhimurium. IFN-γ ^−/−^ mice had higher levels of cecal colonization, and as anticipated, had increased liver CFU at day 3 post-infection (Fig. 5C and D) similar to IL-12p35-deficient mice (Fig 1A and B). Remarkably, IFN-γ^−/−^ mice had substantially reduced cecal inflammation (Fig. 5E, Fig. S3A–D), and even less than p35^−/−^ or p40^−/−^ mice (Fig. 2). The IFN-γ^−/−^ mice also had less injury to the cecal mucosa, as evidence by fewer TUNEL-positive cells and better preservation of Alcian blue positive cells (Fig. 5F and G, and Fig. S3E). The decreased inflammation in IFN-γ^−/−^ mice was associated with fewer CD11b^hi^, CD11c^+^ TNF/MHC class II double positive inflammatory monocytes in the lamina propria, and lower levels of TNF, IL-6 and IL-1β message in the cecal tissue (Fig. S4). There was no difference in IL-17A expression in the cecum (Fig. S4F). These data suggest that IFN-γ is not only important for protection against *Salmonella*, but is also a key regulator of gut inflammation and injury, which is modulated by the activities of IL-23 and IL-12.

### NK1.1+ cells are the primary IFN-γ producers during acute S. Typhimurium infection

**Figure 6:**
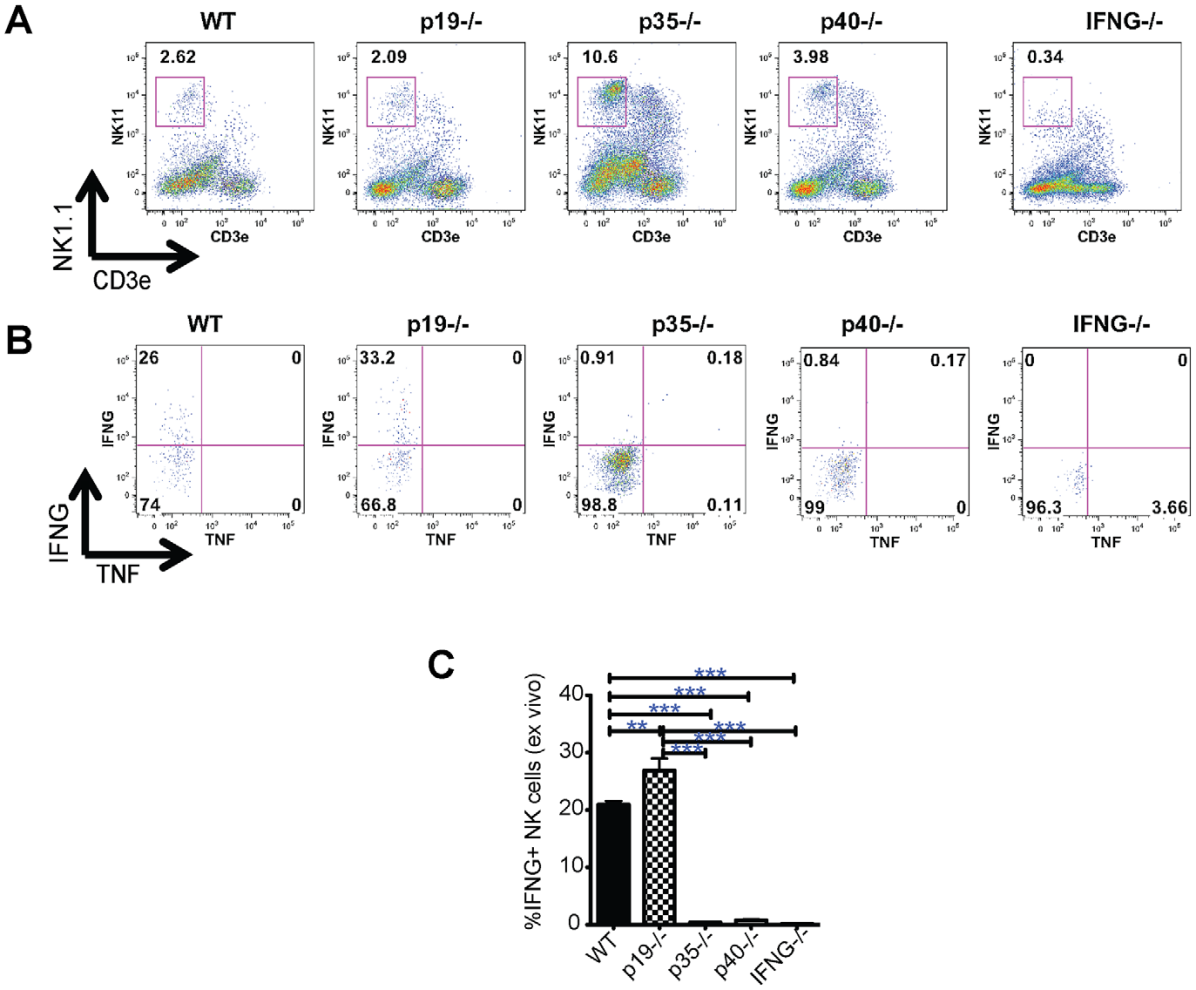
IL-23 inhibits *Salmonella* induced IFN-γ production by lamina propria NK cells. (A) LP populations isolated from WT, p19^−/−^, p35^−/−^, p40^−/−^ and IFN-γ^−/−^ mice were stained for surface expression of NK1.1 and CD3ε; numbers indicate the percentage of cells in the gated box. (B) Intracellular production of IFN-γ and TNF is shown for NK cells (the NK1.1^+^CD3^−^ population gated in A). (C) The percentage of LP IFN-γ+ NK cells were quantified for the aforementioned mice at 3 d *Salmonella* infection. Plotted are the mean ± SE for 3 mice per condition. Statistical significance was determined using the one-way ANOVA followed by Bonferroni post test. *: p<0.05, **: p<0.01, ***: p<0.001

Due to IFN-γ's critical role in acute cecal inflammation, we sought to identify the cell types that produce IFN-γ during *Salmonella* infection. In mock-infected mice, IFN-γ production is essentially undetectable, and stimulation with PMA and ionomycin induces IFN-γ in CD4, CD8, γδ and NK T cells, as well conventional NK cells (Fig. S5A). Upon *S*. Typhimurium infection, all of these subsets demonstrate spontaneous increases in IFN-γ production and NK cells have the highest proportion of lamina propria IFN-γ producing cells (Fig. S5B). Amongst the various mock treated mouse groups, there were no differences in the percentage of NK cells (data not shown). After infection, the percentage of NK cells in the lamina propria decreased in all backgrounds, but p35^−/−^ and p40^−/−^ animals decreased to a lesser degree (Fig. 6A), possibly reflecting an IL-12 dependent dilution of NK cells by recruitment of other cells or loss of NK cells during infection. Both p35^−/−^ and p40^−/−^ NK cells failed to produce IFN-γ *ex vivo* (Figure 6B and C). The loss of IL-23 had no effect on the percentage of NK cells in LP relative to WT mice (Fig 6A), but resulted in a significant increase in IFN-γ production (Fig. 6B and C). Thus in the lamina propria during *Salmonella* infection, IL-12 and IL-23 have opposing roles in regulating IFN-γ by NK cells. Because previous reports have concluded that alveolar and splenic myeloid cells, especially neutrophils, are the primary producers of IFN-γ during acute *S.* Typhimurium infection [27], [28] we examined IFN-γ production by lamina propria neutrophils (Ly6G^hi^ CDllb^hi^) or monocyte/macrophages (Ly6g^int^ CDllb^int^ CDllc^+^) in mock or *S.* Typhimurium infected mice. Lamina propria monocytes and neutrophils showed no specific staining for IFN-γ (Fig. S6A–B). Once again, IFN-γ was produced predominantly by CDllb^int^Ly6g^null^CDllc^null^ cell population, consistent with NK cells (Fig. S6A–B). These data support the role of NK cells as key producers of IFN-γ in the gut following acute *Salmonella* infection.

## Discussion

The mucosal immune system is continuously exposed to trillions of microbes and faced with the daunting task of differentiating benign commensal microbes from detrimental pathogens. This distinction is essential in order to avoid excessive inflammatory responses to commensal organisms and to mount appropriate inflammatory responses against pathogens. Because commensals and pathogens share many if not all of the molecular patterns that are recognized by the innate immune system, additional factors that define pathogenicity, such as invasion and toxicity, must be interpreted by the host at the mucosal surface. Thus benign or mutually beneficial interactions with microbes are non-inflammatory or even immunosuppressive [29], whereas microbes that alter this homeostatic balance trigger inflammation. Innate immune receptors are critical for sensing microbes and regulating inflammatory responses through the production of immunomodulatory cytokines. Several cytokines are essential to maintain tolerance to the gut microbiota, as evidenced by the association of IL-10, FoxP3 or TGFβ1 function in mice or humans with inflammatory bowel disease (IBD) or autoinflammatory disorders [30], [31]. Other cytokines are critical for protection against pathogens that infect hosts through mucosal surfaces. Notably, IL-12/23p40 is essential for proper control of mycobacterial and *Salmonella* infections in humans and mice [5], [6], [4]. IL-12/23p40 and IL-23R have also been associated with mouse and human IBD, respectively, suggesting that these cytokines not only contribute to the proper control of infectious pathogens, but also contribute to excessive and harmful inflammation.

Our studies shed light into the molecular mechanisms that underlie the apparent masking of IL-23 by IL-12 during *Salmonella* infection [5]. Our detailed analyses indicate that IL-23 does not simply have redundant functions that are compensated for by IL-12 during *Salmonella* infection in p19−/− mice. Instead the interaction between IL12 and IL-23 is complex. Loss of IL-23 results in decreased expression of some genes, such as IL-17A and Reg3γ, and increased expression of others such as IL-12p35 and IFN-γ. In the presence of functional IL-12, the loss of IL-23 may not affect susceptibility to *Salmonella* infection due to enhanced IFN-γ production and accumulation of TNF and Nos2 producing cells. However, the compensatory gain in IL-12 and IFN-γ mediated protection against *Salmonella* infection does not go without consequence, and the trade-off is enhanced IL-12 and IFN-γ dependent tissue injury. In the absence of functional IL-12, IL-23 preserves the expression of inflammatory mediators, such as IL-17A, IL-22, Reg3γ, TNF and Nos2, and the accumulation of TNF producing cells in the large intestine of *Salmonella* infected mice.

Our study demonstrates that IL-12 and IL-23 regulate several important inflammatory mediators in the cecum during acute *Salmonella* infection. In particular, both IL-12 and IL-23 contribute to the regulation of MHC class II^+^ TNF producing CD11b^hi^, CD11c^+^ cells. The expression of MHC class II and TNF are also highly dependent on IFN-γ, and our studies indicate that IFN-γ is a major factor for promoting acute *S*. Typhimurium induced intestinal inflammation. Because the intestinal inflammation seen in IFN-γ KO mice was substantially lower than p35^−/−^ and p40^−/−^ mice, IFN-γ appears to also function independent of these cytokines as a critical inducer of early inflammation. IFN-γ is known to trigger release of several CCR-1,-2 and CXCR3 binding chemokines [32] and it is possible that IFN-γ recruits additional inflammatory cells and helps transform the normally immunosuppressive environment of the cecal mucosa into a pro-inflammatory and Th1-biased environment that helps fight *Salmonella* infection. This hypothesis is consistent with the severe reduction in CD11b^hi^, CD11c^+^ cells and NK cells in the cecum of IFN-γ KO mice infected with *Salmonella* (see Fig. 6).

IL-23 regulation of mucosal injury correlates with IL-12 dependent IFN-γ expression; IL-23 suppresses IL-12 expression during mucosal *Salmonella* infection, and thereby limits IFN-γ production. During *Salmonella* infection IL-23 deficiency results in increased IL-12 and IFN-γ production, which is associated with excessive mucosal injury as evidenced by loss of goblet cells and increased cell death within the mucosa. The cell death is most notable in the base of the epithelial crypts, and requires IL-12 and IFN-γ. It is possible that cytokines may be directly toxic to epithelium, or that the cell death may be a manifestation of other IFN-γ regulated factors such as reactive oxygen and nitric oxide. A recent study by Songhet et al. compliments our results and demonstrates that IFNγR contributes to inflammation and epithelial cell injury during acute *Salmonella* infection, and bone marrow chimera studies indicate that IFNγR expression by stromal cells is required for loss of goblet cells seen during acute infection [33]. IFN-γ can act directly on intestinal epithelial cells to influence the β-catenin signaling pathway and epithelial cell homeostasis [34]. Thus during *Salmonella* infection IFN-γ may act directly on epithelial cells, and excessive IFN-γ production may yield epithelial cell injury and death.

In addition to regulating IL-12 and IFN-γ production, our data indicate that IL-23 also has cytoprotective functions, which are mediated at least in part by IL-17RC. IL-17RC^−/−^ mice had increased basal cell death and TUNEL positive cells in the mucosa similar to that observed in p19^−/−^ mice (Fig. 3). IL-17RC is highly expressed on the epithelium of the large intestine [23], [24] and IL-17 has been associated with cytoprotection from inflammation in other models associated with increased IFN-γ production [35], [36]. Administration of α-IL-17A monoclonal antibody had no effect in patients with moderate to severe Crohn's disease, and exacerbated disease in some patients, further suggesting a protective role for IL-17A [37]. Thus IL-17 appears to have important cytoprotective functions in the mucosa, and may be especially important in limiting IFN-γ mediated host cell injury.

IL-6 and IL-10 gene expression are also significantly increased in infected p19−/− mice, and these cytokines may contribute to host defense and mucosal tissue injury against *Salmonella* infection in p19−/−. Upregulation of these cytokines also appears to be dependent on functional IL-12, as seen for IFN-γ. Although we focused our analysis on IFN-γ, IL-6 and IL-10 may also be important mediators of the mucosal responses to *Salmonella* infection. Additional studies will be needed to determine how these factors contribute to host defense, mucosal inflammation and tissue injury during acute *Salmonella* infection.

Our finding that IL-23 cross-regulates IL-12 and modulates IFN-γ is novel to host immune regulation against *Salmonella*, but has been observed in other models. In the TNBS induced colitis model mice deficient in IL-23 produced more IL-12 and were more susceptible to development of colitis than WT mice [38]. In this study, dendritic cells from p19−/− produced elevated levels of IL-12 upon TLR ligation, and treatment of p19−/− mice with an IL-12p40 neutralizing antibody prevented lethal colitis [38]. Similarly, in a tumor initiation and metastasis model, IL-23 suppressed NK cell and IFN-γ dependent anti-tumor activities [39]. Although our data indicate that IL-23 modulates IL-12 expression, IL-23 may also antagonize IL-12 binding to its receptor [40]. Taken together, the data indicate that IL-23 can also negatively impact IFN-γ effector functions and oppose the action of IL-12.

IL-23 also regulates IL-22 production, but during acute *Salmonella* infection, there was only a slight decrease in IL-22 production in p19^−/−^ mice, which was not significant but consistent with prior studies [14]. The IL-22 regulated gene Reg3γ was decreased significantly in the p19^−/−^ mice. There was a significant decrease of IL-22 expression in p40^−/−^ mice but no noticeable difference in p35^−/−^ ceca (Fig. 3), suggesting IL-22 dependence on IL-23 is partially masked by IL-12. Surprisingly, IL-22 deficiency resulted in decreased inflammation (Fig. S2). Thus during acute *Salmonella* infection IL-22 promotes intestinal inflammation, and a portion of this response is regulated independently of IL-23. IL-22 is made by multiple cell types, and in other models of acute intestinal infection and inflammation, innate lymphoid cells appear to be the primary source of IL-22 [13]. In contrast to IFN-γ, we were unable to detect significant levels of IL-22 produced spontaneously in lamina propria cells, suggesting that additional signals mimicked by PMA and ionomycin are needed [41], [42]. It is not known how innate lymphoid cells and NK cells sense infection in the mucosa, and whether they employ innate immune receptors, or respond to changes in cytokines or host cell surface ligands.

In summary we demonstrate that during acute oral *Salmonella* infection, IL-23 suppresses IL-12 and IFN-γ mediated mucosal injury, and, in combination with IL-12, is essential for optimal protection against systemic dissemination. IL-23 contributes to both cytoprotection in the mucosa and host defense, and is critical for fine-tuning the intestinal mucosal inflammatory response in order to limit excessive injury and promote pathogen clearance. Thus cytokines IL-12 and IL-23 work in concert to help respond to infectious pathogens and maintain epithelial homeostasis.

## Methods

### Mice

C57BL/6 (B6), IL-12p35^−/−^, IFN- γ^−/−^ and IL-12p40^−/−^ mice were from The Jackson Laboratory and bred in-house. IL-23p19^−/−^, IL-17RC^−/−^ and IL-22^−/−^ mice bred onto the C57BL/6 background were kindly provided by Dr. Wenjun Ouyang (Genentech) [43], [16]. Information on the generation of C57BL/6 *SLc11a1* ^G169/G169^ congenic mice can be found in Methods S1. Age-matched male and female mice between the ages of 8 and 12 wk were used in experiments. Mice were bred and housed in a specific pathogen-free facility at the University of Washington. All experiments were performed under Institutional Animal Care and Use Committee-approved protocols.

### Bacterial strains

*Salmonella enterica* serovar Typhimurium strain SL1344 [44] was grown overnight at 37°C with shaking (200 rpm) in Luria-Bertani (LB) broth. This strain is resistant to streptomycin.

### Animal experiments

Mice were fasted for 4–6 hr and pretreated with 20 mg of Streptomycin (ISC Bioexpress, Solon, Ohio) in 0.1 cc sterile phosphate buffered saline (PBS) by oral gavage. The following day mice were fasted for 4–6 hr and infected with 1×10^3^ *S.* Typhimurium or nothing (mock) in 0.1 cc of sterile PBS by oral gavage. At 72 hrs after infection, mice were euthanized. Whole cecum and liver were weighed prior to further analysis. To assess bacterial burden sections of each organ were weighed and homogenized in PBS with 0.025% Triton X-100 (EMD chemicals, Darmstadt, Germany), and serial dilutions were plated on streptomycin (50 μg/ml) containing MacConkey agar plates.

### Histopathology/immunohistochemistry

Samples of cecum were fixed overnight at RT in 10% buffered formalin (Sigma-Aldrich, St. Louis, MO), and then processed. Four-micrometer sections were cut and stained with hematoxylin and eosin (H&E) for morphological analysis. Sections were blinded to mouse genotype and evaluated by a board certified anatomic pathologist (KDS). All scoring categories range from 0–3 with increasing level of severity. Scores were assigned for changes to the cecum as follows: **submucosal expansion (S)** – 0 =  no significant change,1 =  <25% of the wall,2 = 25–50% of the wall,3 =  >50% of the wall; **mucosal neutrophilic infiltrate (M)** – 0 =  no significant infiltrate, 1 =  mild neutrophilic inflammation,2 =  moderate neutrophilic inflammation,3 =  severe neutrophilic inflammation; **lymphoplasmacytosis (L)** – 0 =  no significant infiltrate, 1 =  focal infiltrates (mild),2 =  mutifocal infiltrates (moderate),3 =  extensive infiltrates involving mucosa and submucosa (severe); **goblet cells loss (G)** – 0 =  >28/HPF,1 = 11–28/HPF,2 = 1–10/HPF,3 =  <1/HPF. Sections were scored for two other criteria, epithelial integrity and architectural distortion, but these were not affected at 3 d timepoint and thus not included in the final scoring. The combined pathological score for each tissue sample was determined as the sum of these individual scores: 0 no signs of inflammation; 1–3 minimal signs of inflammation that are normally found in untreated specific pathogen-free mice and therefore considered as nonspecific; 4–6 slight inflammation; 6–8 moderate inflammation; >9 profound inflammation. Basal cell death was assessed qualitatively by grading cell death within the basal regions of the crypts as follows: 0 =  no significant dead cells,1 =  focal dead cells,2 =  multifocal cell death, and 3 =  extensive cell death. TUNEL and Alcian Blue staining material and methods can be found in Methods S1. From each group cecal sections from a minimum of 3 different mice were analyzed based on morphology and TUNEL (brown) or Alcian blue (blue) positive cells. At least 10 random high-powered fields (40X objective) were counted from each mouse and means calculated.

### Quantitative real-time PCR (qPCR)

For analysis of changes in gene expression in the mouse cecum after *Salmonella* infection, tissue samples were collected and immediately frozen and stored at −80°C until processing. RNA was then extracted from frozen tissue with TRIzol Reagent (Invitrogen, Carlsbad, CA) according to the instructions of the manufacturer. At least 2 μg of RNA from each sample was reverse transcribed in 20 μl reactions using oligo-dT and the Superscript III reverse transcription reagent (Invitrogen). Real-time PCR was performed on cDNA with TaqMan chemistry (ABI) and commercially available, inventoried probe-primer sets (Applied Biosystems (ABI), San Francisco, CA) according to the manufacturers recommended procedures (ABI). The GAPDH probe-primer set (ABI) was used in all reactions as an internal positive control and to normalize the data. Real-time PCR was performed using and ABI 7900 real-time PCR system.

### Preparation of lamina propria (LP) cells

To prepare single cell suspensions, large bowel (cecum and colon) were resected, and contents purged with PBS. The isolation of lamina propria cells was adapted from Lugering et al. [45], and is described in detail in Methods S1. LP cells were counted using Trypan Blue exclusion to measure viability (>95% viability) and approximately 1×10^5^–10^6^ cells/well were distributed in 96 well round bottom plates. LP cell suspensions from mock or infected animals were cultured for approximately 4 hrs with 10 ug/ml Brefeldin A (Sigma) at 37°C in 96-well round bottom plates prior to staining for flow cytometry. In some experiments, cells were also stimulated with PMA (50 ng/ml) and ionomycin (0.7 μM) for 4 hours in the presence of BFA.

### Flow cytometry

All antibodies used for flow cytometric analysis were purchased from BD Biosciences (San Jose, CA) or eBioscience (San Diego, CA). Following incubation, cells were treated with 2 mM EDTA in PBS for 10 min at 37°C to detach adherent cells. All future steps were incubated on ice. Cells were washed with PBSA (1% w/v BSA in PBS), and then Fc receptors were blocked using α-CD16/32 for 15 min. Surface and intracellular staining material and methods can be found in Methods S1. We collected 1–3x10^5^ gated events using a BD LSR II or Canto I flow cytometer. The data were analyzed using FlowJo software (Treestar, Inc., Ashland, OR).

### Statistics

We calculated statistical significance with Prism software (GraphPad, La Jolla, CA). Unless otherwise specified, all studies for which data are presented are representative of at least two independent experiments. The D'Agostino & Pearson omnibus normality test confirmed that the log transformed gene expression and CFU data passed the normality test. Statistical analysis of more than two normally distributed groups was performed using ANOVA with Bonferroni post-test; comparison of two normally distributed groups was done using the unpaired Student's T-test with Welch's correction for unequal variance. For multiple comparisons of non-Gaussian distributed data, the groups were analyzed using the Kruskal–Wallis test followed by Dunn's post-test, and comparison of two groups of non-Gaussian distributed data was performed using the Mann–Whitney rank-sum test. Statistical significance was defined as a *P* value <0.05, and indicated in the figure legends.

## Supporting Information

Methods S1 **Supplemental methods for this article.**
(DOCX)

Figure S1 **The role of IL-12 and IL-23 in protection against systemic CFU is independent of Slc11a1 genotype.** *Slc11a1* ^G169/G169^ congenic WT, p19^−/−^ and p35^−/−^, mice were orally infected with 1x10^3^ CFU of *S*. Typhimurium 1 d after pretreatment with 20 mg streptomycin. Bacterial burden was determined for (A) Cecum and (B) liver from mice at 3 days post infection. Bars represent the median bacterial load and the data are pooled from 2 separate infections. Statistical significance was determined using one way ANOVA with the Bonferroni post test ***: p<0.001.
(TIF)

Figure S2 **IL-22 promotes cecal inflammation and injury following** ***Salmonella*** **infection.** (A) Cecum and (B) liver *Salmonella* CFU from IL-22^−/−^ and C57BL/6 mice. Bars represent the median bacterial load. (C) Scoring of inflammatory changes at 72 hr p.i. from infected WT and IL-22^−/−^ B6 mice. (D)TUNEL positive cells were quantified per high-power field in the ceca of at minimum 3 infected mice from each group. (E) IL-22, (F)Reg3γ, (G)IFN-γ and (H) TNF cecal gene expression data at 3 d after oral *Salmonella* infection from WT and IL-22^−/−^ mice. Data are expressed as the ratio of mRNA levels of the gene of interest divided by GAPDH expression from the same RNA. Statistical significance was determined using the unpaired Student's t-test. *: p<0.05, **: p<0.01, ***: p<0.001.
(TIF)

Figure S3 **IFN-γ is required for** ***Salmonella*** **induced colitis.** (A) Representative ceca from WT and IFN-γ^−/−^ at 3 d following *Salmonella* infection or mock treatment. The bar equals 1 cm. (B) Low and (C) high power H&E stained cecal sections of WT and IFN-γ^−/−^ mice at 3 d after infection. Arrows in (C) indicate dead cells. (D) TUNEL-positive (brown) and (E) Alcian Blue (AB)-PAS-stained (blue) cells of representative infected cecal sections. Bar in (B) represents 200 microns and (C–E) 50 microns.
(TIF)

Figure S4 **IFN-γ is essential for** ***Salmonella*** **-induced activation of inflammatory monocytes and pro-inflammatory cytokine expression.** (A) LP CDllb^hi^/CDllc^+^ populations generated from C57BL/6, and IFN-γ^−/−^ mice were assayed for their MHC class II expression and intracellular production of TNF. (B) TNF, (C) IL-6, (D) IL-1β, (E) IFN-γ, and (F) IL-17A gene expression in cecal tissue was measured by qPCR. Data are expressed as the ratio of mRNA levels of the gene of interest divided by GAPDH expression from the same RNA. Statistical significance was determined using the unpaired Student's t-test. *: p<0.05, **: p<0.01, ***: p<0.001.
(TIF)

Figure S5 **NK cells have the largest proportion and number of LP IFN-γ producing cells among LP populations 3 d following** ***Salmonella*** **infection.** 3 d lamina propria cells from (A) mock or (B) *Salmonella* infected C57BL/6 mice were stimulated for 6 hr with or without PMA and ionomycin in the presence of BFA. Cells were then stained for surface markers: CD3ε, CD8, CD4, NK1.1, then fixed, permeabilized and stained for intracellular IFN-γ.
(TIF)

Figure S6 **LP monocytes and neutrophils are not major producers of IFN-γ.** After 3 d mock or *Salmonella* infection LP cells were isolated from C57BL/6 and IFN-γ^−/−^ mice, and treated BFA for 6 hr (A) or left untreated (B). Cells were stained for CDllb, CDllc, and Ly6G, then permeabilized and stained for IFN-γ (top panels) or IgG1 isotype control (bottom panels). Green histogram: Mock C57BL/6; Blue histogram: Infected IFN-γ^−/−^; Red histogram: Infected C57BL/6. Histograms are from pooled cells of 2 mice per group.
(TIF)
