# Supplementary material for: Homeostatic Regulation of Salmonella-Induced Mucosal Inflammation and Injury by IL-23
Source: PLoS One. 2012 May 18;7(5):e37311. doi: 10.1371/journal.pone.0037311 (PMC3356277; doi:10.1371/journal.pone.0037311)
Supplement: Methods S1 — Supplemental methods for this article. (DOCX) [file pone.0037311.s001.docx]

**Supplemental Methods:**

**Mice:** C57BL/6 mice have an inactivating G169D mutation in Nramp1, whereas A/J mice have the functional allele that transmits resistance to *S*. Typhimurium, *M. bovis* BCG and *Leishmania* infections ^1^. The gene encoding Nramp1 (Slc11a1) is located on mouse chromosome 1. We generated C57BL/6 *SLc11a1*^G169/G169^ congenic mice by crossing C57BL/6 mice with a line of C57BL/6 mice consomic for A/J chromosome 1 (C57BL/6J-Chr 1^A/J^/NaJ, The Jackson Laboratory), and selecting offspring for optimal crossover events with Illumina murine SNP chips (Ilumina). After several generations of backcrossing, we derived heterozygous mice with A/J *SLc11a1*^G169/G169^ allele flanked by 3 Mb from A/J chromosome 1, and all other chromosomes genotyped as C57BL/6. These mice were bred to homozygosity at *Slc11a1*^G169/G169^, and are genetically 99.8% C57BL/6. The p19^-/-^, p35^-/-^, and IL-17RC^-/-^ mice were backcrossed onto the *Slc11a1*^G169/G169^ congenic C57BL/6 mice.

**TUNEL/AB IHC:** Apoptotic cells were stained by the TUNEL method as previously described^46^, on cecal paraffin sections using an Apoptag peroxidase *in situ* apoptosis detection kit (Millipore, Billerica, MA). Control slides were processed identically, but without the TdT. In order to stain for the acidic mucin positive goblet cells, paraffin embedded cecal sections were first rehydrated then stained with Alcian blue (Sigma). Lithium carbonate (Sigma), saffron (Sigma) and H&E were used for counterstains prior to dehydration for each section.

**Lamina Propria Isolation:** Intestines were cut open lengthwise into 1cm pieces and washed 2X with CMF solution [10 mM HEPES, 25 mM sodium bicarbonate (Sigma), and 2% FBS (Atlas Biological, Fort Collins, CO) in Ca^2+^, Mg^2+^-free HBSS (Invitrogen), pH 7.2]. Tissue was transferred into EDTA/DTT/FBS/CMF solution [1.3 mM EDTA (Invitrogen), 1mM DTT (VWR, Radnor, PA), 10% FBS, in CMF supplemented with 1X HGPG (5 mM HEPES, 2 mM L-glutamine (Fisher Scientific), 20 U/ml penicillin/20 ug/ml streptomycin (Fisher Scientific) and20 ug/ml gentamicin (Invitrogen) )]. After 20-min shaking at 250 rpm in 37°C for 20 min, samples were vortexed for 10s and epithelial cells from the supernatant were discarded. This step was repeated followed by two washes in CMF and the tissue was filtered through nylon mesh (70 μm nylon, Becton Dickinson (BD), San Jose, CA). LP suspensions were prepared from the EDTA treated de-epithelialized tissue by further incubation with collagenase solution [0.5 mg/ml of type II collagenase (Worthington, Lakewood, NJ), 1X HGPG and 5% FBS] at 37°C while shaking (250rpm) for 1 hr. The LP cells were isolated from the interface of a 40/80 Percoll gradient after centrifugation for 20 min at 2,400 rpm at RT. Cells were washed twice with supplemented RPMI 1640 medium [100 U/ml penicillin/100 ug/ml streptomycin, 100 ug/ml gentamicin (Invitrogen), 10% FBS, and 50uM β- mercaptoethanol (Sigma)].

**Flow Cytometry:** For myeloid surface markers cells were stained with α-CD11c (FITC or PE-CY7), α-CDllb (PERCP-Cy5.5), α-I-A^b^ (FITC), and Ly6G (APC). For lymphoid surface markers cells were stained with α-CD3ε (APC), α-CD4 (PERCP), α-CD8 (PERCP), α-γδ TCR (FITC) and α-NK1.1(PE). Cells were washed with PBSA and resuspended in cytoperm/cytofix solution (BD) for 20 min on ice and then washed with Perm Wash (BD). For intracellular staining, myeloid sets were stained with α-TNF (Pacific Blue) and α-IL-6 (PE), while the lymphoid sets were stained with α-IFN-γ (PE-Cy7), α-TNF (Pacific Blue) and α-IL-17a (PE or FITC). Stained cells were washed several times with Perm Wash solution and resuspended in fresh 1% PFA solution

**Reference:**

1. Blackwell, J. M. et al. SLC11A1 (formerly NRAMP1) and disease resistance. *Cell Microbiol* **3**, 773-784 (2001).
