# Supplementary figures and images for: Homeostatic Regulation of Salmonella-Induced Mucosal Inflammation and Injury by IL-23
Source: PLoS One. 2012 May 18;7(5):e37311. doi: 10.1371/journal.pone.0037311 (PMC3356277; doi:10.1371/journal.pone.0037311)

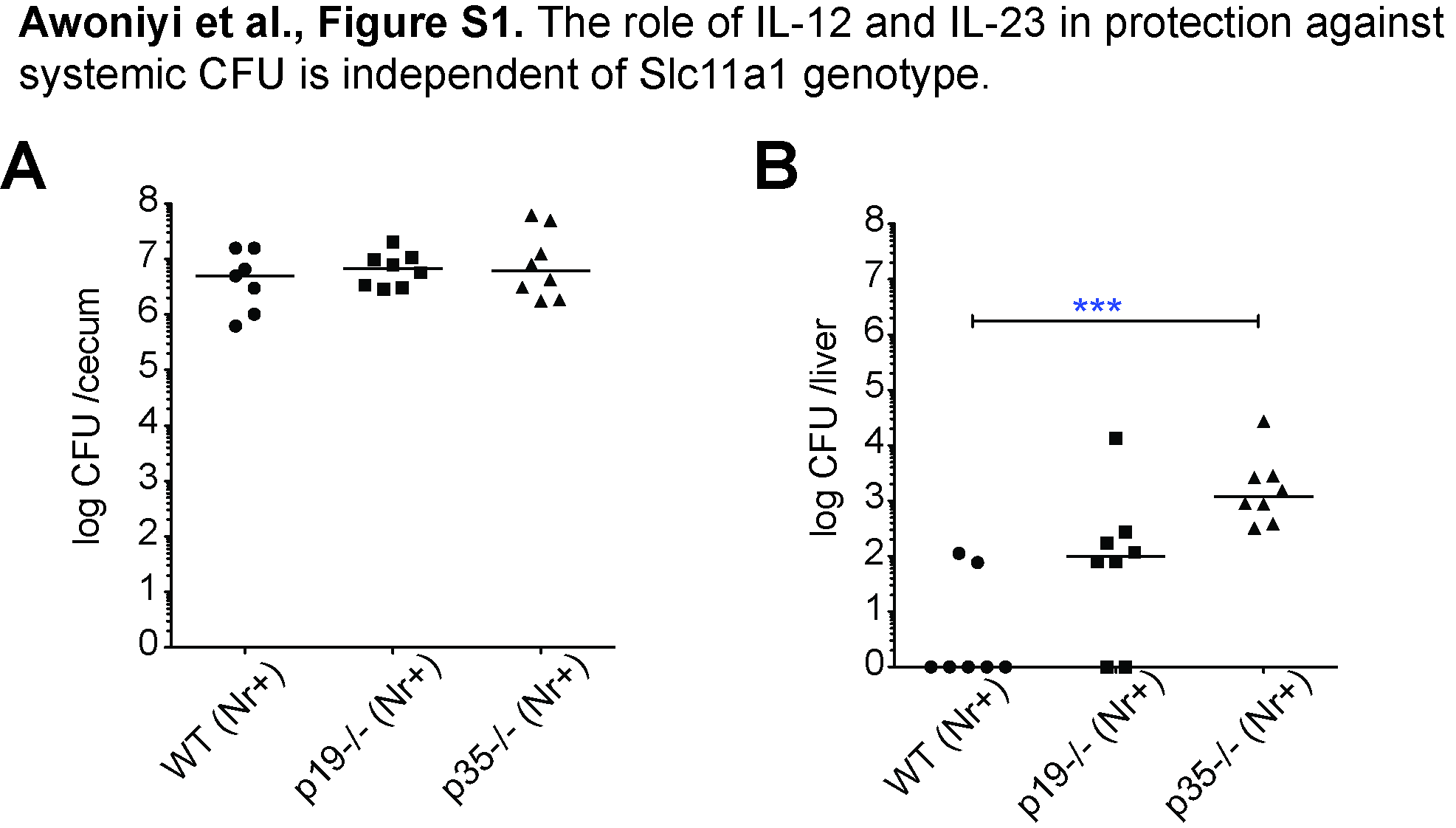

Supplement: Figure S1 — The role of IL-12 and IL-23 in protection against systemic CFU is independent of Slc11a1 genotype. Slc11a1 G169/G169 congenic WT, p19−/− and p35−/−, mice were orally infected with 1x10^3^ CFU of S. Typhimurium 1 d after pretreatment with 20 mg streptomycin. Bacterial burden was determined for (A) Cecum and (B) liver from mice at 3 days post infection. Bars represent the median bacterial load and the data are pooled from 2 separate infections. Statistical significance was determined using one way ANOVA with the Bonferroni post test ***: p<0.001. (TIF) [file pone.0037311.s002.tif]

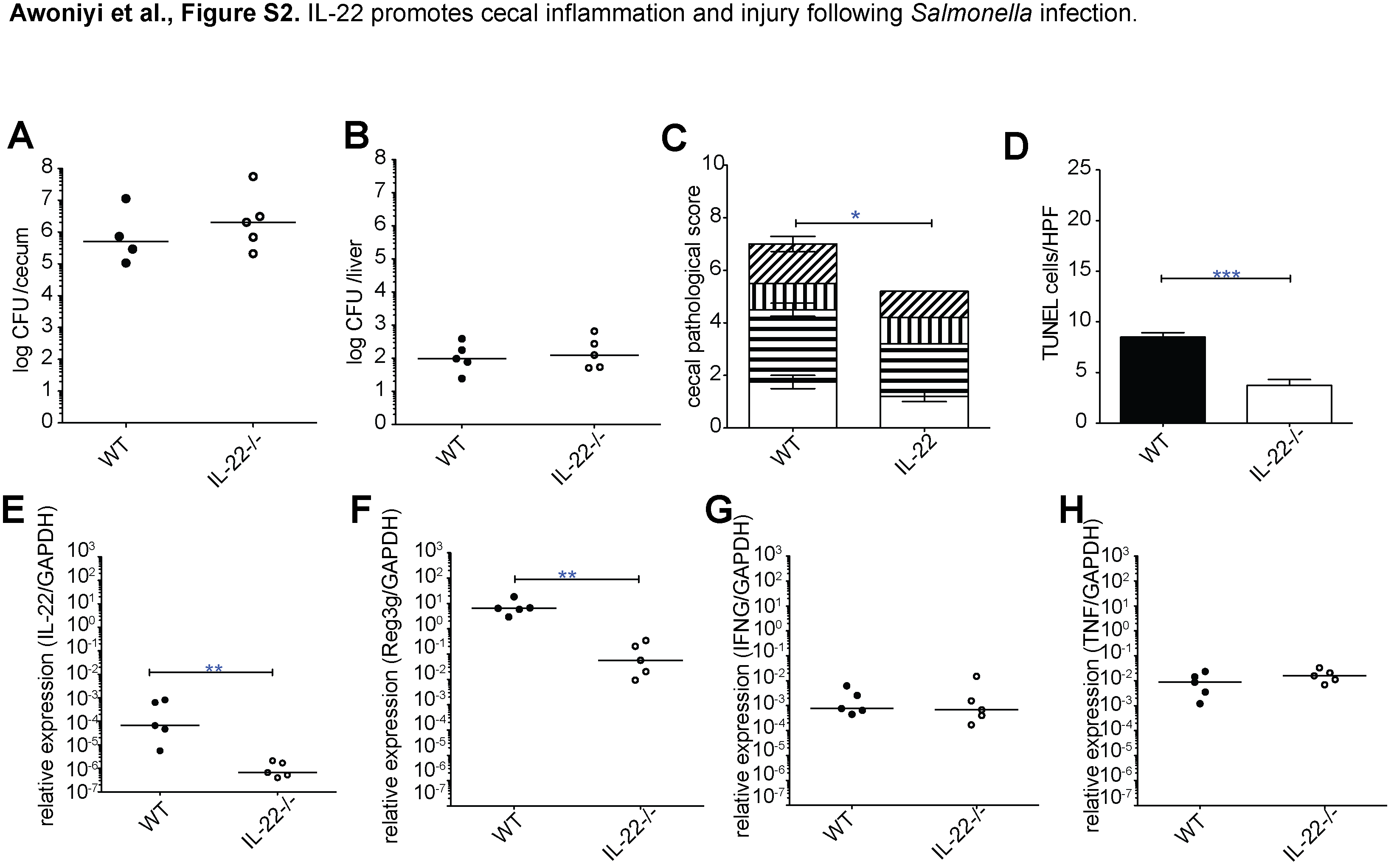

Supplement: Figure S2 — IL-22 promotes cecal inflammation and injury following Salmonella infection. (A) Cecum and (B) liver Salmonella CFU from IL-22−/− and C57BL/6 mice. Bars represent the median bacterial load. (C) Scoring of inflammatory changes at 72 hr p.i. from infected WT and IL-22−/− B6 mice. (D)TUNEL positive cells were quantified per high-power field in the ceca of at minimum 3 infected mice from each group. (E) IL-22, (F)Reg3γ, (G)IFN-γ and (H) TNF cecal gene expression data at 3 d after oral Salmonella infection from WT and IL-22−/− mice. Data are expressed as the ratio of mRNA levels of the gene of interest divided by GAPDH expression from the same RNA. Statistical significance was determined using the unpaired Student's t-test. *: p<0.05, **: p<0.01, ***: p<0.001. (TIF) [file pone.0037311.s003.tif]

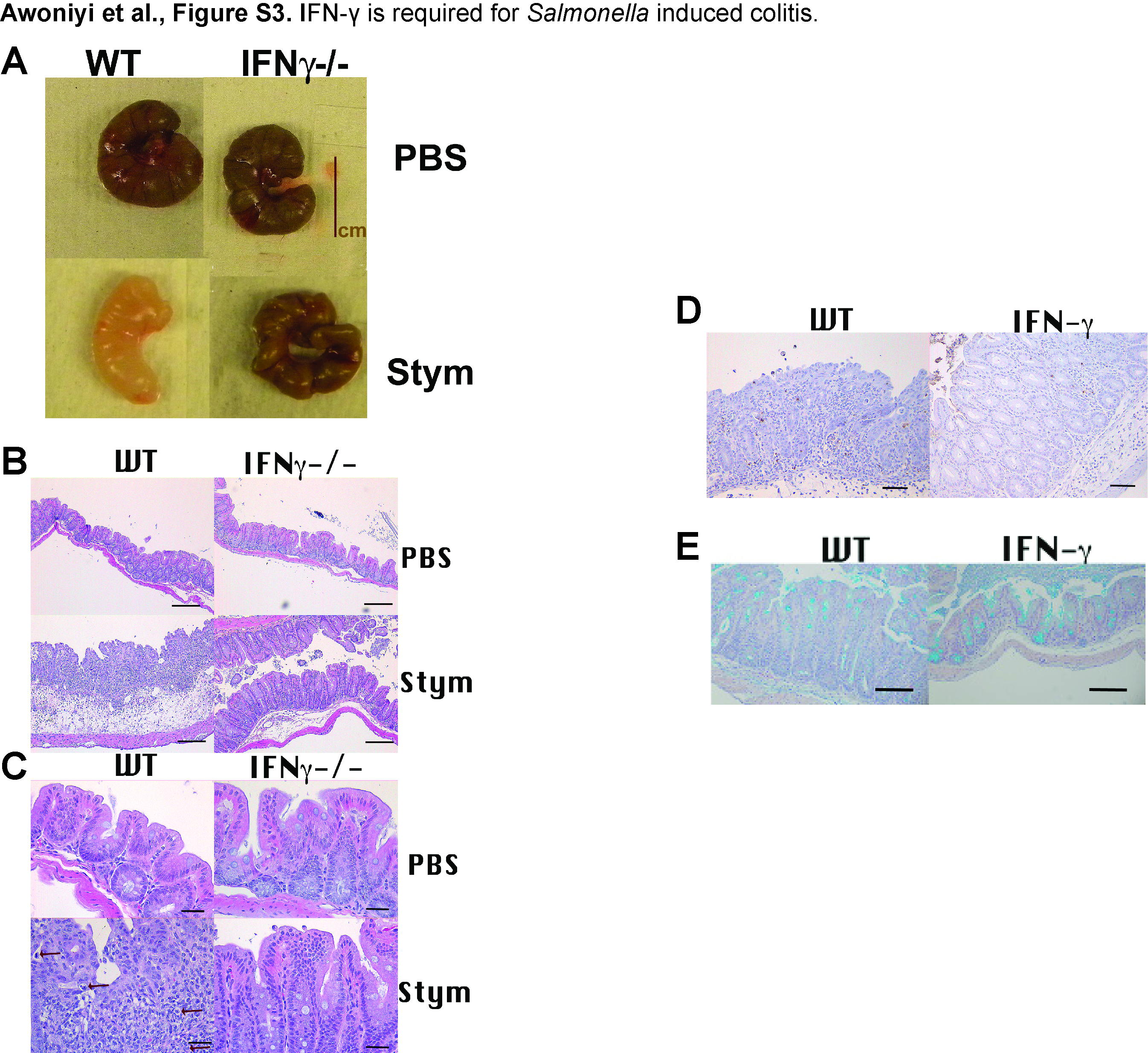

Supplement: Figure S3 — IFN-γ is required for Salmonella induced colitis. (A) Representative ceca from WT and IFN-γ−/− at 3 d following Salmonella infection or mock treatment. The bar equals 1 cm. (B) Low and (C) high power H&E stained cecal sections of WT and IFN-γ−/− mice at 3 d after infection. Arrows in (C) indicate dead cells. (D) TUNEL-positive (brown) and (E) Alcian Blue (AB)-PAS-stained (blue) cells of representative infected cecal sections. Bar in (B) represents 200 microns and (C–E) 50 microns. (TIF) [file pone.0037311.s004.tif]

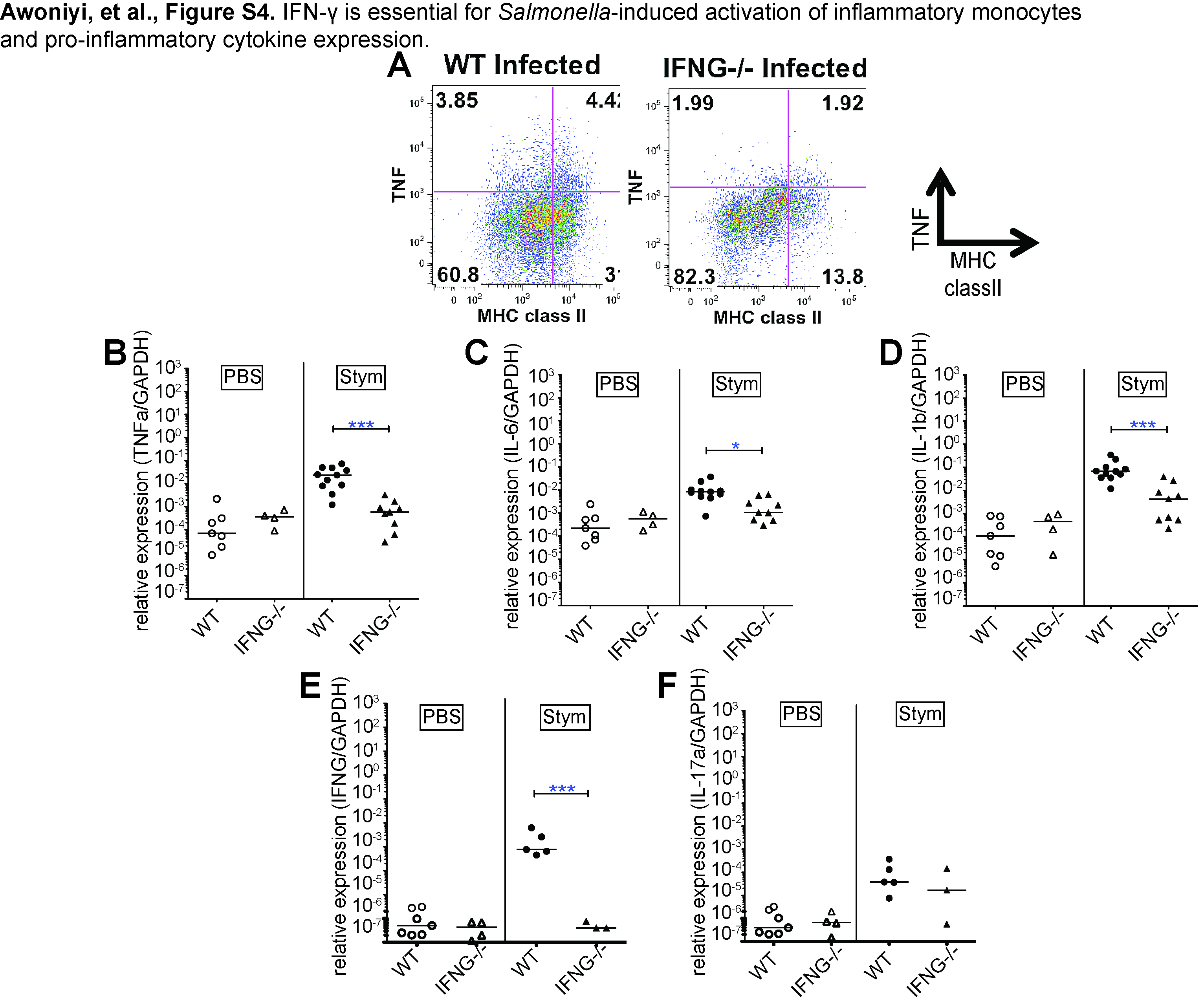

Supplement: Figure S4 — IFN-γ is essential for Salmonella -induced activation of inflammatory monocytes and pro-inflammatory cytokine expression. (A) LP CDllbhi/CDllc+ populations generated from C57BL/6, and IFN-γ−/− mice were assayed for their MHC class II expression and intracellular production of TNF. (B) TNF, (C) IL-6, (D) IL-1β, (E) IFN-γ, and (F) IL-17A gene expression in cecal tissue was measured by qPCR. Data are expressed as the ratio of mRNA levels of the gene of interest divided by GAPDH expression from the same RNA. Statistical significance was determined using the unpaired Student's t-test. *: p<0.05, **: p<0.01, ***: p<0.001. (TIF) [file pone.0037311.s005.tif]

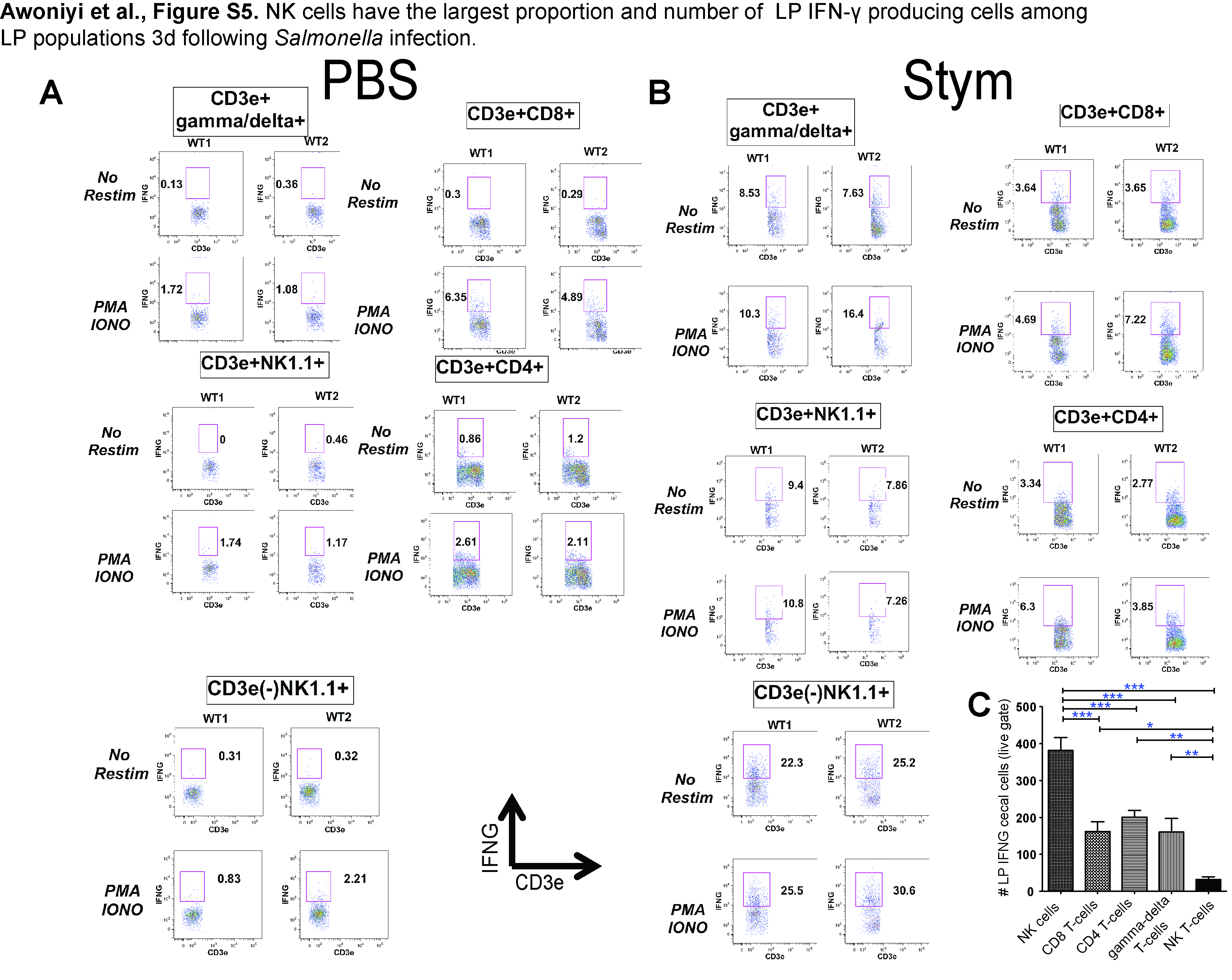

Supplement: Figure S5 — NK cells have the largest proportion and number of LP IFN-γ producing cells among LP populations 3 d following Salmonella infection. 3 d lamina propria cells from (A) mock or (B) Salmonella infected C57BL/6 mice were stimulated for 6 hr with or without PMA and ionomycin in the presence of BFA. Cells were then stained for surface markers: CD3ε, CD8, CD4, NK1.1, then fixed, permeabilized and stained for intracellular IFN-γ. (TIF) [file pone.0037311.s006.tif]

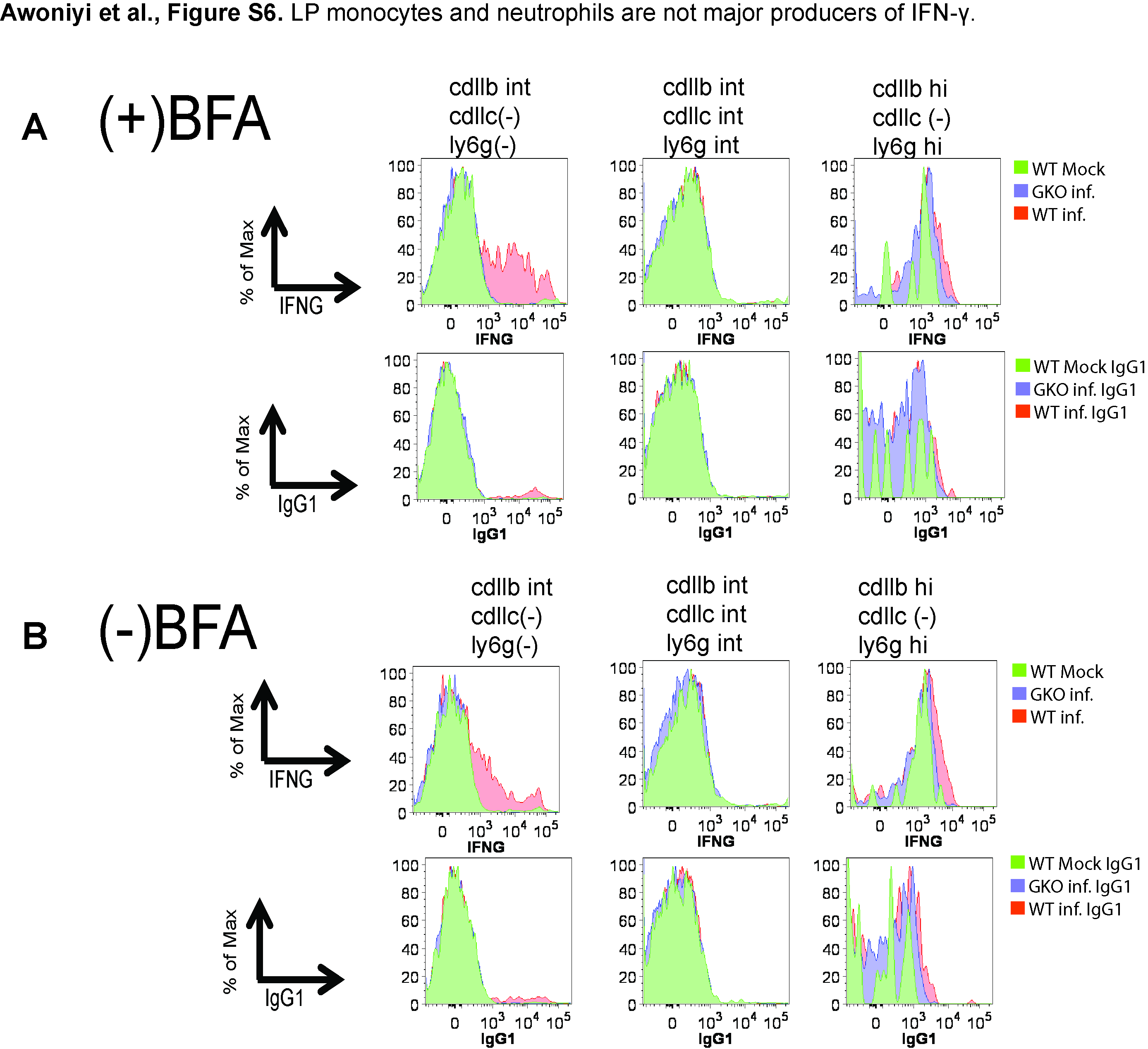

Supplement: Figure S6 — LP monocytes and neutrophils are not major producers of IFN-γ. After 3 d mock or Salmonella infection LP cells were isolated from C57BL/6 and IFN-γ−/− mice, and treated BFA for 6 hr (A) or left untreated (B). Cells were stained for CDllb, CDllc, and Ly6G, then permeabilized and stained for IFN-γ (top panels) or IgG1 isotype control (bottom panels). Green histogram: Mock C57BL/6; Blue histogram: Infected IFN-γ−/−; Red histogram: Infected C57BL/6. Histograms are from pooled cells of 2 mice per group. (TIF) [file pone.0037311.s007.tif]
